# Comparing CAM Algorithms for the Identification of Salient Image Features in Iconography Artwork Analysis

**DOI:** 10.3390/jimaging7070106

**Published:** 2021-06-29

**Authors:** Nicolò Oreste Pinciroli Vago, Federico Milani, Piero Fraternali, Ricardo da Silva Torres

**Affiliations:** 1 Department of Electronics Information and Bioengineering, Politecnico di Milano, 20133 Milano, Italy; nicolooreste.pinciroli@mail.polimi.it (N.O.P.V.); piero.fraternali@polimi.it (P.F.); 2 Department of ICT and Engineering, NTNU—Norwegian University of Science and Technology, 6009 Ålesund, Norwayricardo.torres@ntnu.no (R.d.S.T.)

**Keywords:** convolutional neural network, class activation maps, explainability, iconography, artwork analysis

## Abstract

Iconography studies the visual content of artworks by considering the themes portrayed in them and their representation. Computer Vision has been used to identify iconographic subjects in paintings and Convolutional Neural Networks enabled the effective classification of characters in Christian art paintings. However, it still has to be demonstrated if the classification results obtained by CNNs rely on the same iconographic properties that human experts exploit when studying iconography and if the architecture of a classifier trained on whole artwork images can be exploited to support the much harder task of object detection. A suitable approach for exposing the process of classification by neural models relies on Class Activation Maps, which emphasize the areas of an image contributing the most to the classification. This work compares state-of-the-art algorithms (CAM, Grad-CAM, Grad-CAM++, and Smooth Grad-CAM++) in terms of their capacity of identifying the iconographic attributes that determine the classification of characters in Christian art paintings. Quantitative and qualitative analyses show that Grad-CAM, Grad-CAM++, and Smooth Grad-CAM++ have similar performances while CAM has lower efficacy. Smooth Grad-CAM++ isolates multiple disconnected image regions that identify small iconographic symbols well. Grad-CAM produces wider and more contiguous areas that cover large iconographic symbols better. The salient image areas computed by the CAM algorithms have been used to estimate object-level bounding boxes and a quantitative analysis shows that the boxes estimated with Grad-CAM reach 55% average IoU, 61% GT-known localization and 31% mAP. The obtained results are a step towards the computer-aided study of the variations of iconographic elements positioning and mutual relations in artworks and open the way to the automatic creation of bounding boxes for training detectors of iconographic symbols in Christian art images.

## 1. Introduction

Iconography is the discipline that concerns itself with the subject matter of artworks, as opposed to their form [1]. It is studied to understand the meaning of artworks and to analyze the influence of culture and beliefs on art representations across the word, from the Nasca [2] to the Byzantine [3] civilization. Iconography is a prominent topic of the art history studied through centuries [4,5,6]. The attribution of iconographic elements (henceforth *classes*) is an important task in art history, related to the interpretation of meaning and to the definition of the geographical and temporal context of an artwork.

With the advent of digital art collections, iconographic class attribution has acquired further importance, as a way to provide a significant index on top of digital repositories of art images, supporting both students and experts in finding and comparing works by their iconographic attributes. However, the analysis of iconography requires specialized skills, based on the deep knowledge of the symbolic meaning of a very high number of elements and of their evolution in space and time. The WikiPedia page on Christian Saint symbolism (https://en.wikipedia.org/wiki/Saint_symbolism—accessed on 15 May 2021) lists 257 characters with 791 attributes. This makes the manual attribution of iconographic classes to image collections challenging, due to the tension between the available amount of expert work and the high number of items to be annotated.

A viable alternative relies on the use of semi-automatic computer-aided solutions supporting the expert annotator in the task of associating iconographic classes to art images. Computer Vision (CV) has already been used for artwork analysis tasks, such as genre identification [7], author identification [8], and even subject identification and localization [9]. The field of computer-aided iconographic analysis is more recent and addressed by few works [10,11]. Borrowing the standard CV terminology, the problem of computer-aided iconographic analysis can be further specialized into iconography classification, which tackles the association of iconographic classes to an artwork image as a whole, and iconography detection, which addresses the identification of the regions of an image in which the attributes representing an iconographic class appear.

Applying CV to the analysis of art iconographic poses challenges, in part, general and, in part, specific to the art iconography field. As in general-purpose image classification and object detection, the availability of large high quality training data is essential. The natural image dataset in use today are very large and provided with huge numbers of annotations. Conversely, in the narrower art domain, image datasets are less abundant, smaller, and with less high-quality annotations. Furthermore, unlike natural images, painting images are characterized by less discriminative features than natural ones. The color palette is more restricted and subject to artificial effects, such as colored shadows and chiaroscuro. Images of paintings may also portray partially deteriorated subjects (e.g., in frescoes) and belong to historical archives of black and white photos.

Despite the encouraging results of applying Convolutional Neural Networks (CNNs) for iconography classification [11], it remains unclear how such a task is performed by artificial models. Depending on the class, the human expert may consider the whole scene portrayed in the painting or instead focus on specific hints. Considering Christian art iconography, an example of the first scenario occurs in paintings of complex scenes such as the crucifixion or the visitation of the magi. The latter case is typical of the identification of characters, especially Christian saints, which depends on the presence of very distinctive attributes. When CNNs are used for the classification task, the problem of explainability arises, i.e., of exposing how the CNN has produced a given result. A widely used strategy to clarify CNN image classification results relies on the use of Class Activation Maps [12,13,14], which visualize the regions of the input images that have the most impact on the prediction of the CNN. Computing the most salient regions of an image with respect to its iconography can help automate the creation of bounding boxes around the significant elements of an artwork from image-wide annotations only. This result could reduce the effort of building training sets for the much harder task of iconography detection.

This paper addresses the following research questions:

- Are CAMs an effective tool for understanding how a CNN classifier recognizes the iconographic classes of a painting?
- Are there significant differences in the state-of-the-art CAM algorithms with respect to their ability to support the explanation of iconography classification by CNNs?
- Are the image areas highlighted by CAMs a good starting point for creating semi-automatically the bounding boxes necessary for training iconography detectors?

**Figure 1:**
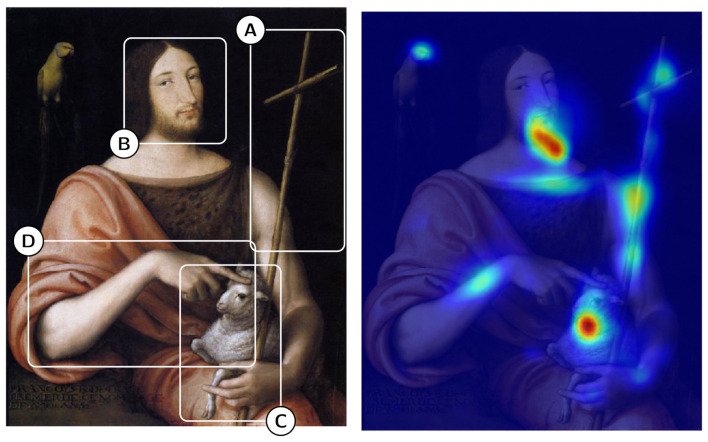
On the left: Saint John the Baptist image and iconographic symbols identified manually (e.g., cross (**A**), face (**B**), and lamb (**C**), and hand pointing at lamb (**D**)). On the right: the CAM heat map associated with classification results of a CNN-based solution.

The contributions of the paper can be summarized as follows:

- We apply four state-of-the-art class activation map algorithms (namely, CAM [15], Grad-CAM [16], Grad-CAM++ [17], and Smooth Grad-CAM++ [18]) to the CNN iconography classification model presented in [11], which exploits a backbone based on ResNet50 [19] trained on the ImageNet dataset [20] and refined on the ArtDL dataset (http://www.artdl.org—accessed on 15 May 2021) consisting of 42,479 images of artworks portraying Christian Saints divided into 10 classes. Note that, in order to avoid ambiguity, we refer to the specific algorithm as “CAM” and to the generic output as “class activation maps”.
- For the quantitative evaluation of the different algorithms, a test dataset has been built which comprises 823 images annotated with 2957 bounding boxes surrounding specific iconographic symbols. One such annotated image is shown in Figure 1. We use the Intersection over Union (IoU) metrics to measure the agreement between the areas of the image highlighted by the algorithm and those annotated manually as ground truth. Furthermore, we analyze the class activation map area based on percentage of covered bounding boxes and percentage of covered area that does not contain any iconographic symbol.
- The comparison shows that Grad-CAM, Grad-CAM++, and Smooth Grad-CAM++ deliver better results than the original CAM algorithm in terms of area coverage and explainability. This finding confirms the result discussed in [18] for natural images. Smooth Grad-CAM++ produces multiple disconnected image regions that identify small iconographic symbols quite precisely. Grad-CAM produces wider and more contiguous areas that cover well both large and small iconographic symbols. To the best of our knowledge, such a comparison has not been performed before in the context of artwork analysis.
- We perform a qualitative evaluation by examining the overlap between the ground-truth bounding boxes and the class activation maps. This investigation illustrates the strengths and weaknesses of the analyzed algorithms, highlights their capacity of detecting symbols that were missed by the human annotator and discusses cases of confusion between the symbols of different classes. A simple procedure is tested for selecting “good enough” class activation maps and for creating symbol bounding boxes automatically from them. The results of such a procedure are illustrated visually.
- We deepen the evaluation by measuring quantitatively the agreement between the ground-truth bounding boxes and the bounding boxes estimated from the class activation maps. The assessment shows that the whole Saint bounding boxes computed from the Grad-CAM class activation maps obtain 55% average IoU, 61% GT-known localization and 31% mAP. Such results obtained by a simple post-processing of the output of a general purpose CNN interpretability technique pave the way to the use of automatically computed bounding boxes for training weakly supervised object detectors in artwork images.

Figure 1 shows an example of the assessment performed in this paper. On the left, an image of Saint John the Baptist has been manually annotated with the regions (from A to D) associated with key symbols relevant for iconography classification. On the right, the same image is overlaid with the CAM heat map showing the regions contributing the most to the classification.

The rest of the paper is organized as follows: Section 2 surveys related work; Section 3 describes the different CAM variants considered in our study; Section 4 describes the adopted evaluation protocol and the results of the quantitative and the qualitative analysis; finally, Section 5 draws the conclusions and outlines possible future work.

## 2. Related Work

This section surveys the essential previous research in the fields of automated artwork analysis and CNN interpretability that are the foundations of our work.

### 2.1. Automated Artwork Image Analysis

The large availability of artworks in digital format has allowed researchers to perform automated analysis in the fields of digital humanities and cultural heritage by means of Computer Vision and Deep Learning methods. Several datasets containing various types of artworks have been proposed to support such studies [10,11,21,22,23,24,25,26].

The performed analyses span several classification tasks and techniques: from style classification to artist identification, comprising also medium, school, and year classification [27,28,29]. These researches are useful to support cultural heritage studies and asset management, e.g., automatic cataloguing of unlabeled works in online and museum collections, but their results can be exploited for more complex applications, such as authentication, stylometry [30], and forgery detection [31].

A task that is more related to our proposal is artwork content analysis, which focuses on the automatic identification and, if possible, localization of objects inside artworks. The literature contains several state-of-the-art approaches [9,10,11,32,33,34,35]. Since there is abundance of deep learning models trained with natural images but a deficiency of art-specific models, many studies focus on the transferability of previous knowledge to the art domain [11,35,36,37,38]. This approach is known as Transfer Learning and consists in fine-tuning a network, previously trained with natural images, using art images. The consensus is that Transfer Learning is beneficial for tasks related to artworks analysis.

### 2.2. Interpretability and Activation Maps

In recent years, Deep Learning models have been treated as black-boxes, i.e., architectures that do not expose their internal operations to the user. These systems are used for various approaches and their interpretability is fundamental in many fields, especially when the outputs of the models are used for sensitive applications. In the literature, there are many techniques that aim at explaining the behavior of neural models [39,40]. Saliency Masks are used to address the outcome explanation problem by providing a visualization of which part of the input data is mainly responsible for the network prediction. The most popular Saliency Masks are obtained with the Class Activation Map (CAM) approach. CAMs [15] have shown their effectiveness in highlighting the most discriminative areas of an image in several fields, ranging from medicine [41] to fault diagnostics [12]. The original formulation of CAMs has been subsequently improved. Selvaraju et al. [16] introduced Grad-CAM, which exploits the gradients that pass through the final convolutional layer to compute the most salient areas of the input. Chattopadhay et al. [17] introduced Grad-CAM++ which considers gradients too but is based on a different mathematical formulation that improves the localization of single and multiple instances. Smooth Grad-CAM++ [18] applies Grad-CAM++ iteratively on the combination of the original image and a Gaussian noise.

The use of CAMs is not limited to the explainability of Deep Learning classification models but is the starting point for studies related to the weakly supervised localization of content inside the images [42].

This paper focuses on the comparison of different CAM algorithms on the task of iconography classification to determine which variant may be more suitable for weakly supervised studies. Since CAM algorithms are most often studied only for natural images, the aim of the work is also to address the research gap about the utility of CAMs for the art domain.

## 3. Class Activation Maps for Iconography Classification

This paper compares different CAM algorithms: Grad-CAM, Grad-CAM++, and Smooth Grad-CAM++. Their implementation is based on the mathematical definitions provided, respectively, by [15,16,17,18].

**Figure 2:**
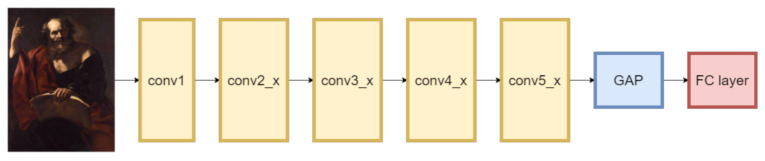
The ResNet50 architecture.

Figure 2 shows the ResNet50 classifier architecture used to compute the class activation maps. The input of the network is an image and the output is the set of probabilities associated with the different classes. In the evaluation, the input images portray art works and the output classes denote 10 Christian Saints. ResNet50 contains an initial convolutional layer (conv1) followed by a sequence of convolutional residual blocks (conv2_x …conv5_x). A Global Average Pooling (GAP) module computes the average value for each feature map obtained as an output of the last layer (conv5_x). The probability estimates are computed by the last component, which is typically a fully connected (FC) layer [43].

### 3.1. CAM

CAMs [15] are based on the use of GAP, which has been demonstrated to have remarkable localization abilities [44]. The GAP operation averages the feature maps of the last convolutional layer and feeds the obtained values to the final fully connected layer that performs the actual classification. Class activation maps are generated by performing a weighted sum of the feature maps of the last convolutional layer for each class. The actual class activation map value $M_{c}\left(x,y\right)$ for a class *c* and a position x,y in the input image is expressed as follows:

(1) $$M_{c}\left(x,y\right)=\sum_{k}w_{k}^{c}A_{k}\left(x,y\right)$$

where $A_{k}\left(x,y\right)$ is the activation value of feature map *k* in the last convolutional layer at position (x,y), and $w_{c}^{k}$ is the weight associated with feature map *k* and with class *c*. Intuitively, a high CAM value at position x,y is the result of an average high activation value of all the feature maps of the last convolutional layer.

Differently from the original approach, we compute the CAM output not only for the predominant class, but for all the classes. The ArtDL dataset contains multi-class multi-label images and this formulation allows us to analyze which regions of the artwork are associated with which classes, also in the case of wrong classification.

### 3.2. Grad-CAM

Grad-CAM [16] is a variant of CAM which considers not only the weights but also the gradients flowing into the last convolution layer. In this way, also the layers preceding the last one contribute to the activation map. An advantage of using gradients is that Grad-CAM can be applied to any layer of the network. Still, the last one is especially relevant for the localization of the parts of the image that contribute most to the final prediction. Furthermore, the layer used as input for the prediction can be followed by any module and not only by a fully connected layer. Grad-CAM exploits the parameters $\alpha_{k}^{c}$, which represents the neuron importance weights and are calculated as:

(2) $$\alpha_{c}^{k}=\frac{1}{Z}\sum_{i}\sum_{j}\frac{\partial y^{c}}{\partial A_{ij}^{k}}$$

where $\frac{1}{Z}\sum_{i}\sum_{j}$ denotes the global average pooling operation (Z=i·j) and $\frac{\partial y^{c}}{\partial A_{ij}^{k}}$ denotes the back-propagation gradients. In the gradient expression, $y^{c}$ is the score of the class *c* and $A^{k}$ represents the *k*-th feature map. The Grad-CAM for a class *c* at position (x,y) is then given by:

(3) $$M_{Grad-CAM}^{c}\left(x,y\right)=ReLU\left(\sum_{k}\alpha_{c}^{k}A^{k}\left(x,y\right)\right)$$

where the ReLU operator maps the negative values to zero. As in the case of CAM, we compute the output of Grad-CAM for all the classes under analysis.

### 3.3. Grad-CAM++

Grad-CAM++ [17] is a generalization of Grad-CAM aimed at better localizing multiple class instances and at capturing objects more completely. Differently from Grad-CAM, Grad-CAM++ applies a weighted average of the partial derivatives, with the purpose of covering a wider portion of the object. Given a class *c* with a score $Y^{c}$ and the activation map $A_{ij}^{k}$ calculated in the last convolutional layer, a parameter $\alpha_{ij}^{kc}$ can be defined as follows:

(4) $$\alpha_{ij}^{kc}=\frac{\frac{\partial^{2}Y^{c}}{{\left(\partial A_{ij}^{k}\right)}^{2}}}{2\frac{\partial^{2}Y^{c}}{{\left(\partial A_{ij}^{k}\right)}^{2}}+\sum_{a}\sum_{b}A_{ab}^{k}\left\{\frac{\partial^{3}Y^{c}}{{\left(\partial A_{ij}^{k}\right)}^{3}}\right\}}$$

The parameter $w_{k}^{c}$, which has the same role of $\alpha_{k}^{c}$ in Grad-CAM, is defined as:

(5) $$w_{k}^{c}=\sum_{i}\sum_{j}\alpha_{ij}^{kc}ReLU\left(\frac{\partial Y^{c}}{\partial A_{ij}^{k}}\right)$$

which leads to

(6) $$w_{k}^{c}=\sum_{i,j}\left[\frac{\frac{\partial^{2}Y^{c}}{{\left(\partial A_{ij}^{k}\right)}^{2}}}{2\frac{\partial^{2}Y^{c}}{{\left(\partial A_{ij}^{k}\right)}^{2}}+\sum_{a,b}A_{ab}^{k}\left\{\frac{\partial^{3}Y^{c}}{{\left(\partial A_{ij}^{k}\right)}^{3}}\right\}}\right]ReLU\left(\frac{\partial Y^{c}}{\partial A_{ij}^{k}}\right)$$

As in the other CAMs, it holds that

(7) $$M_{Grad-CAM++}^{c}\left(x,y\right)=ReLU\left(\sum_{k}w_{k}^{c}A^{k}\left(x,y\right)\right)$$

### 3.4. Smooth Grad-CAM++

Smooth Grad-CAM++ [18] is a variant of Grad-CAM++ that can focus on subsets of feature maps or of neurons for identifying anomalous activations. Smooth Grad-CAM++ applies random Gaussian perturbations on the image *z* and exploits the visual sharpening of the class activation maps by averaging random samples taken from a feature map close to the input. The value of the activation map $M_{c}$ in a position (x,y) is defined as:

(8) $$M_{c(x,y)}\left(z\right)=\frac{1}{n}\sum_{1}^{n}M_{c(x,y)}^{GCpp}\left(z+N\left(0,\sigma^{2}\right)\right)$$

where *n* is the number of samples, $N(0,\sigma^{2})$ is the 0-mean Gaussian noise with standard deviation σ, and $M_{c}^{SGCpp}$ is the activation map for the input $z+N(0,\sigma^{2})$. The final result is obtained by iterating the computation of Grad-CAM++ on inputs resulting from the overlap of the original image and a random Gaussian noise.

## 4. Evaluation

**Figure 3:**
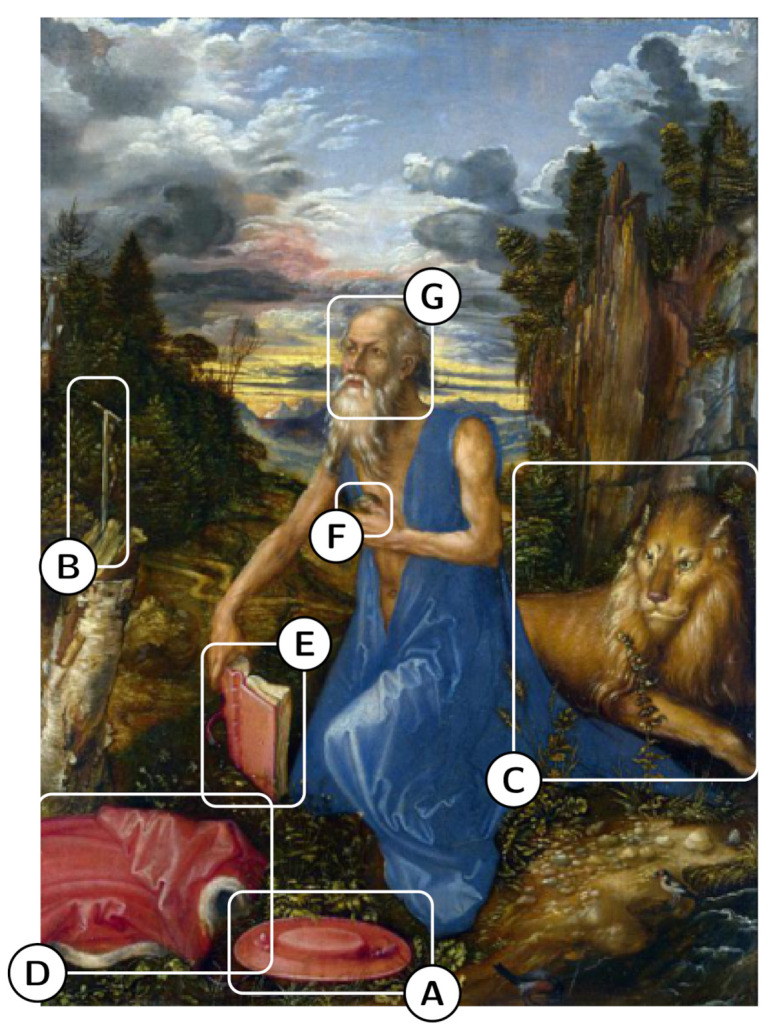
**Saint Jerome**—The cardinal’s galero (**A**), the crucifix (**B**), the lion (**C**), the cardinal’s vest (**D**), the book (**E**), the stone in the hand (**H**), and the face (**G**).

The evaluation exploits the ArtDL dataset [11], an existing artwork collection annotated with image-level labels. The purpose of the evaluation is: (1) to understand whether the class activation maps are effective in localizing both the whole representation of an iconographic class and the distinct symbols that characterize it (the attributes associated with the classes present in the ArtDL dataset are illustrated in [45] and listed in [46]); (2) to compare CAMs algorithms in their ability to do so. To evaluate the localization ability of class activation maps, a subset of the images have been annotated with bounding boxes framing iconographic symbols associated with each Saint. Figure 3 illustrates the symbols in a painting of Saint Jerome. The bounding boxes are used for the quantitative assessment of class activation maps algorithms with the metrics described in Section 4.5. A qualitative analysis is reported in Section 4.6.

### 4.1. Dataset

The ArtDL dataset [11] comprises images of paintings that represent the Iconclass [47] categories of 10 Christian Saints: Saint Dominic, Saint Francis of Assisi, Saint Jerome, Saint John the Baptist, Saint Anthony of Padua, Saint Mary Magdalene, Saint Paul, Saint Peter, Saint Sebastian, and the Virgin Mary. The representation of such classes in Christian art paintings exploit specific symbols, i.e., markers that hint at the identity of the portrayed character. Table 1 summarizes the symbols associated with the 10 Iconclass categories represented in the ArtDL dataset.

The ArtDL images are associated with high-level annotations specifying which Iconclass categories appear in them (from a minimum of 1 to a maximum of 7). Whole-image labels are not sufficient to assess the different ways in which the class activation maps methods focus on the image content. For this purpose, it is necessary to annotate the dataset with bounding boxes that localize the symbols listed in Table 1. Out of the whole dataset, 823 sample images were selected and manually annotated with bounding boxes that frame each symbol separately. A symbol can either be included completely within a single bounding box (e.g., Saint Jerome’s lion) or be split into multiple bounding boxes (e.g., Saint Peter’s bushy hair, which are usually divided in two parts separated by the forehead). We consider a symbol representation as the union of all the bounding boxes annotated with the same symbol label. For instance, Saint Sebastian’s arrows correspond to a unique symbol but are annotated with multiple bounding boxes. When the same symbol relates to multiple saints (e.g., Baby Jesus may appear with both the Virgin Mary and St. Anthony of Padua), its presence is denoted with a label composed of the the symbol name and the Saint’s name. While some symbols appear in the majority of the images of the corresponding Saint, others are absent or rarely present. For each Saint, only the symbols that appear in at least 5% of the paintings depicting the respective Saint are kept. This filter eliminates 23 of the 84 possible symbols associated with the 10 Iconclass categories and reduces the number of symbol bounding boxes from 2957 to 2887. Table 2 summarizes the characteristics of the dataset used to compare the class activation maps algorithms.

**Figure 4:**
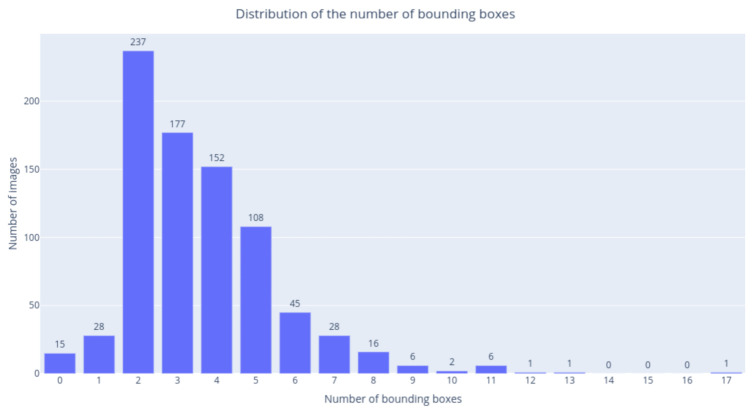
Bounding box distribution: most images contain from 2 to 5 bounding boxes (average = 3).

Figure 4 shows the distribution of the bounding boxes within the images. Most images contain from 2 to 5 bounding boxes and a few images do not contain any annotation. The latter case occurs when the automatic classification of the ArtDL dataset is incorrect (e.g., for images in which a character named Mary was incorrectly associated with the Virgin Mary).

### 4.2. Class Activation Maps Generation

The class activation maps are generated by feeding the image to the ResNet50 model and applying the computation explained in Section 3. They have a size equal to h×w×c where *h* and *w* are the height and width of the *conv5_x* layer and *c* is the number of classes. Since the output size (*h*, *w*) is smaller than the input size, due to the convolution operations performed by the ResNet architecture, each class activation map is upsampled with bilinear interpolation to match the input image size. Min-max scaling is applied to the upsampled class activation maps to normalize them in the [0,1] range.

### 4.3. Choice of the Threshold Value

**Figure 5:**
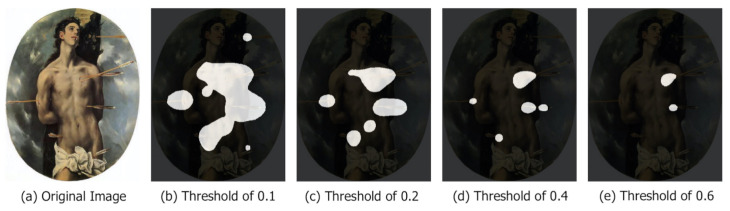
Analysis with different thresholds—black areas correspond to class activation map values below the specified threshold (background) while white pixels correspond to class activation map values greater or equal than the threshold (foreground). An increment in the threshold value results in smaller and more distinct areas. Original image (**a**), cam with threshold at 0.1 (**b**), cam with threshold at 0.2 (**c**), cam with threshold at 0.4 (**d**), cam with threshold at 0.6 (**e**).

A class activation map contains values in the range from 0 to 1. Given a threshold *t*, it is possible to separate the class activation map into background (pixels with a value lower than *t*) and foreground (pixels with a value greater than *t*). The choice of the threshold value aims at making foreground areas concentrate on the Saints’ figure and symbols. Figure 5 shows the impact of applying different threshold values to a class activation map. As the threshold value increases, the foreground areas (in white) become smaller and more distinct and the background pixels increase substantially at the cost of fragmenting the foreground areas and missing relevant symbols. To investigate the choice of the proper threshold, the quantitative evaluation of Section 4.5 reports results obtained with multiple values uniformly distributed from 0 to 1 with a step of 0.05.

### 4.4. Intersection Over Union Metrics

Intersection Over Union (IoU) is a standard metric used to compute the overlap between two different areas. It is defined as:

$$IoU=\frac{A_{\cap }}{A_{\cup }},$$

where $A_{\cap }$ is the intersection between the two areas and $A_{\cup }$ is their union. IoU ranges between 0 and 1, with 0 meaning that the two areas are disjoint and 1 meaning that the two areas overlap and have equal dimensions. We use IoU to compare the foreground regions of the class activation maps with the ground-truth bounding boxes. The computation of the class activation maps and of the metrics does not depend on the number of Saints in the painting, because every Iconclass category is associated with a different activation map independent of the others. All the reported results are valid regardless of the number of Saints.

### 4.5. Quantitative Analysis

This section presents the results of comparing quantitatively the effectiveness of the class activation maps algorithms in the localization of iconography classes and their symbols.

Smooth Grad-CAM++ is the only method that requires hyper-parameters: the standard deviation σ and the number of samples *s*. To set the hyper-parameter values, a grid-search was executed in the following space: σ∈{0.25,0.5,1} and s∈{5,10,25}. Only the best and worst Smooth Grad-CAM++ configurations are reported, to emphasize the boundary values reached by this algorithm. The number of samples is found to barely affect the results, whereas the standard deviation has more impact. To reduce the computational cost a lower number of samples is preferable.

Component IoU

This metric evaluates how well the class activation map focuses on the individual Saints’ symbols. First, the class activation map foreground area is divided into connected components, i.e., groups of pixels connected to each other. The IoU value is calculated between each ground-truth bounding box and the connected components that intersect it. Then, the average IoU across all symbol classes is taken. This procedure is repeated for all threshold values.

**Figure 6:**
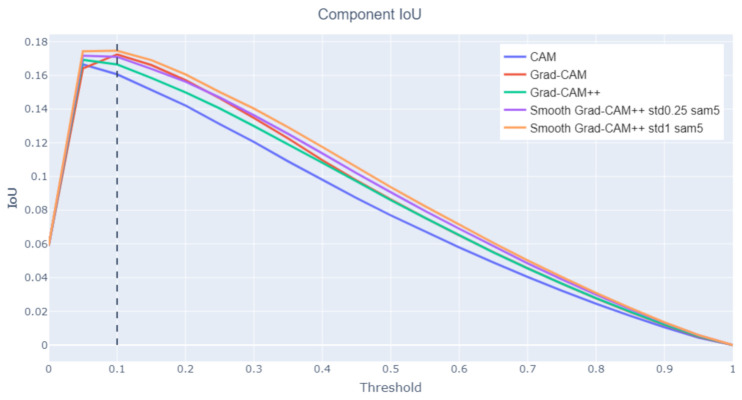
Component IoU at varying threshold levels.

**Figure 7:**
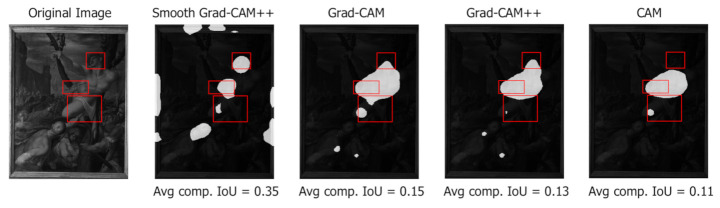
Different values of component IoU produced by different class activation map algorithms (Smooth Grad-CAM++ with σ=1 and s=5) at threshold t=0.1. Ground-truth bounding boxes are shown in red.

Figure 6 shows that the best results are obtained by Smooth Grad-CAM++ with a standard deviation σ=1 and a number of samples s=5. The reason for this is that Smooth Grad-CAM++ tends to produce smaller and more focused areas, which yield more connected components and better coverage of the distinct symbols. Grad-CAM tends to create larger and more connected areas. This increases the size of the union and such an increase is not compensated by an equivalent increase of the intersection, which motivates the lower IoU values. In all the considered class activation maps variants, the component IoU peak is found for a threshold value t∈{0.05,0.1}. Grad-CAM creates larger and more connected regions, and thus, a higher threshold is needed to obtain the same number of components as the other methods. This explains why the component IoU peak is found at a higher threshold. Figure 7 (San Sebastian’s Martyrdom, Giovanni Maria Butteri, 1550–1559) compares the component IoU values produced on a sample image by different class activation maps algorithms. For the same threshold value, Smooth Grad-CAM++ creates more and better focused components.

Global IoU

**Figure 8:**
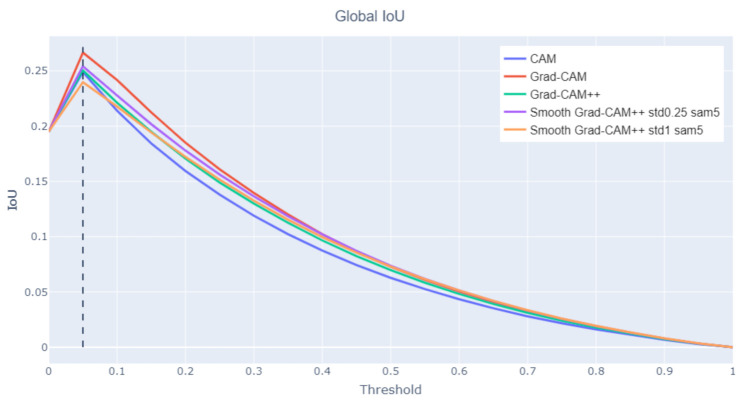
Global IoU at varying threshold levels.

**Figure 9:**
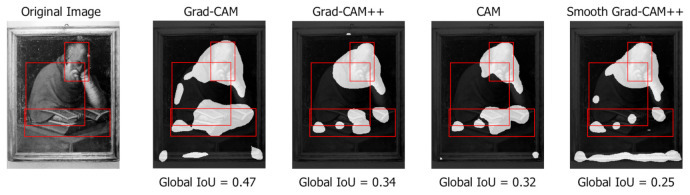
Different values of global IoU produced by different class activation map algorithms (Smooth Grad-CAM++ with σ=1 and s=5) at threshold t=0.05. Manually annotated symbol bounding boxes are shown.

An alternative metric is the IoU between the union of all the bounding boxes in the image and the entire foreground area of the class activation map taken at a given threshold. This metric is calculated for all threshold values and assesses how the class activation map focuses on the whole representation of the Saint, favoring those class activation maps methods that generate wider and more connected areas rather than separated components. Figure 8 shows that Grad-CAM is significantly better than the other analyzed methods. As already observed, Grad-CAM tends to spread over the entire figure and covers better the Saint and the associated symbols. Due to the complementary role of the component and global IoU metrics, the method with the best component IoU (Smooth Grad-CAM++ with σ=1 and s=5) has the worst global IoU. Differently from the component IoU, the global IoU peak position on the *x* axis does not change across methods, because the influence of the number of components is less relevant when the global metric is computed. Figure 9 (Saint Jerome in the study, nd, 1604) compares the global IoU values produced on a sample image by different class activation map algorithms. For the same threshold value, Grad-CAM generates wider areas that cover more foreground pixels.

Bounding box coverage 

**Figure 10:**
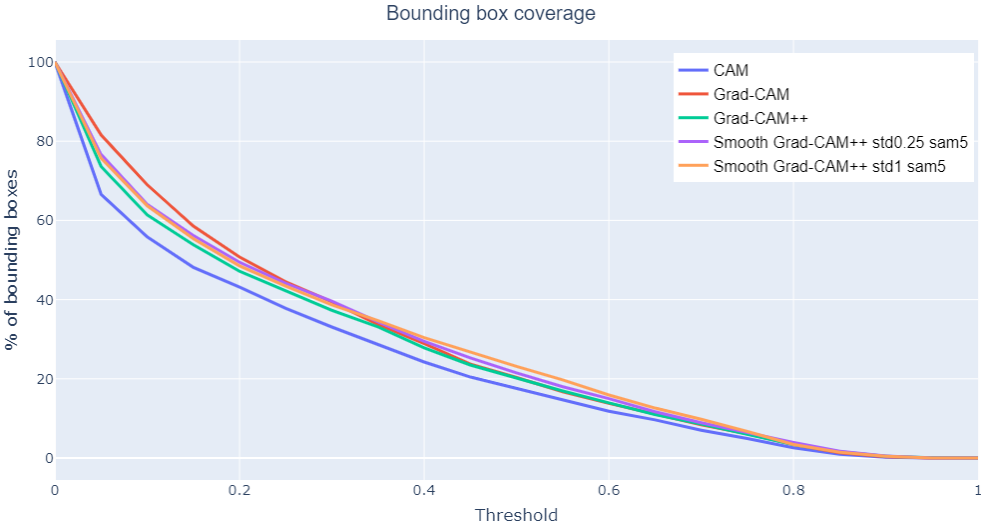
Bounding box coverage at varying threshold values.

When analyzing the class activation map algorithms, a factor to consider is also how many bounding boxes are covered by each class activation map. This metric alone is not enough to characterize the performance because a trivial class activation map that covers the entire image would have 100% coverage. However, coupled with the two previous metrics, it can give information about which method is able to generate class activation maps that can highlight a large fraction of the iconographic symbols that an expert would recognize. The bounding box coverage metric considers that a bounding box is covered by the class activation map only if their intersection is greater than or equal to 20% of the bounding box area. Figure 10 presents the results: Grad-CAM and Smooth Grad-CAM++ intersect, on average, more bounding boxes than the other methods. This result confirms that Grad-CAM covers wider areas, while focusing on the correct details at the same time. The worst method, CAM, performs poorly also in the two previous metrics. This indicates that it generates class activation maps that are smaller and less focused on the iconographic symbols with respect to the other approaches.

Irrelevant attention

**Figure 11:**
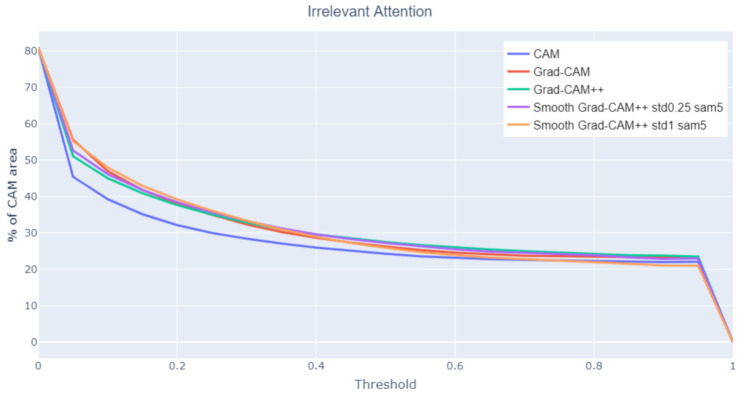
Irrelevant attention at varying threshold values.

**Figure 12:**
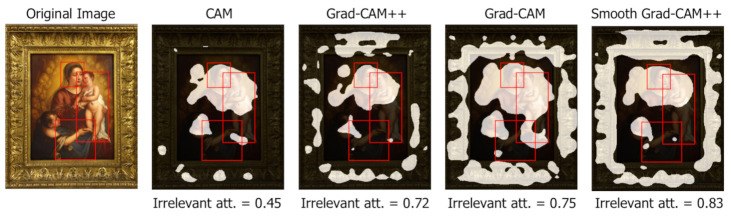
Different values of irrelevant attention produced by different class activation map algorithms (Smooth Grad-CAM++ with σ=1 and s=5) at threshold t=0.1. Manually annotated symbol bounding boxes are reported.

When evaluating the global IoU, a low value can occur for two reasons: (1) the two areas have a very small intersection or (2) the two areas overlap well but one is much larger than the other. Thus, an analysis on how much the class activation maps focus on irrelevant parts of the image helps characterizing low global IoU values. Irrelevant attention corresponds to the percentage of class activation map area outside any bounding box. Figure 11 shows that CAM has the less irrelevant attention, coherently with the previous results. Figure 12 (Madonna with Child and Infant St. John surrounded by Angels, Tiziano Vecellio, 1550) compares the irrelevant attention values produced on a sample image by different class activation map algorithms. For the same threshold value, CAM generates smaller irrelevant areas whereas Grad-CAM and Smooth-Grad-CAM++ include more irrelevant regions corresponding to the painting frame. The tendency of Smooth Grad-CAM++ to focus on irrelevant areas can be seen also in Figure 7 and Figure 9.

### 4.6. Qualitative Analysis

This section presents a qualitative analysis of the results obtained by the different class activation map algorithms highlighting their capabilities and limitations. Each example shows the original image, the class activation maps generated by each algorithm (with background in black and foreground in white) and the ground-truth bounding boxes.

Positive examples

**Figure 13:**
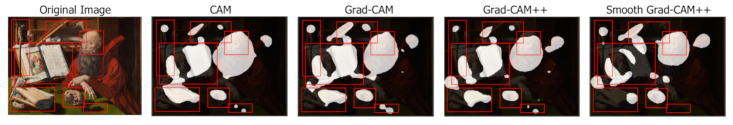
Class activation maps with seven recognized symbols associated with Saint Jerome.

Figure 13 (Saint Jerome in his Study, Jan van Remmerswale, 1533) shows an example in which all the algorithms focus well on the iconographic symbols. The image contains seven symbols with different size, shape and position, which are all identified and separated by the class activation map algorithms. The irrelevant area on the top right corresponds to a piece of the cardinal’s vest that has the same color and approximate shape of the cardinal’s galero appearing in many paintings of Saint Jerome.

**Figure 14:**
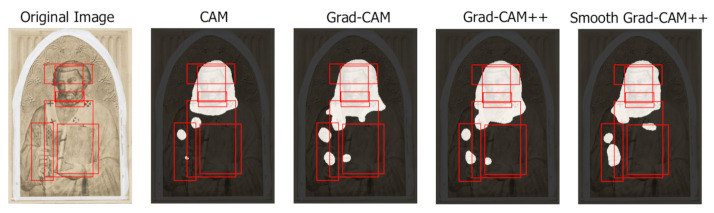
Class activation maps extracted from a drawing of Saint Peter. Four out of five symbols are identified despite their low visibility.

Figure 14 (St. Peter, Antonio Veneziano, 1369–1375) shows an example in which all the algorithms perform well on a painting in which the visibility of the symbols is very low. All class activation map algorithms identify four out of the five symbols. The central ground-truth bounding box is not identified because it corresponds to a rather generic attribute (the bishop’s vest), which is not evident in the drawing. Only CAM misses the book, which the other algorithms identify by focusing on the characteristic marks on the spine of the book or on the lock. The example of Figure 14 and many similar ones of black and white and poor quality images highlight the ability of class activation map algorithms to extract useful maps also when the image has low discriminative features.

**Figure 15:**
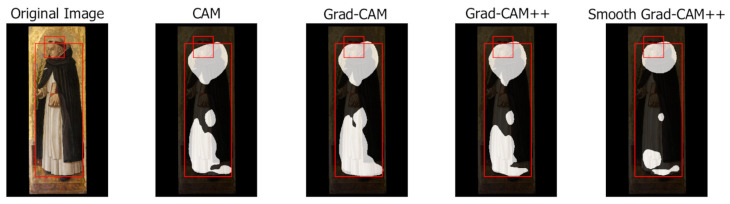
Class activation maps extracted from a paining of Saint Dominic. The rather generic vest attribute is identified by focusing on its double color.

A counterexample of the difficulty of detecting such generic attributes as the vest is illustrated in Figure 15 (Saint Dominic, Carlo Crivelli, 1472). The vest is identified thanks to a specific detail: the change of color typical of the black and white Dominican habit.

Negative examples

**Figure 16:**
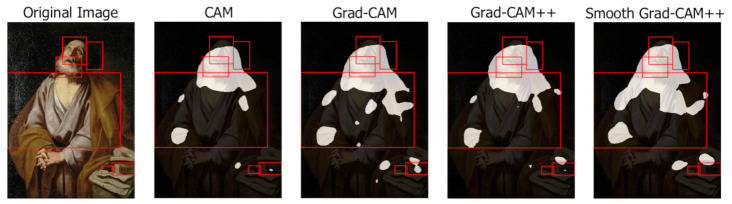
Class activation maps with merged symbols and missed generic attributes.

Class activation maps algorithms tend to fail consistently in two cases: when multiple symbols are too close or have a substantial overlap and when the representation of a symbol is rather generic and covers a wide area of the image. Figure 16 (Penitent St. Peter, Jusepe de Ribera, 1600–1649) illustrates a typical example: Saint Peter’s bushy hair and beard are merged into a single region and the vest, which is a rather generic attribute, is missed completely or highlighted only through small irrelevant details.

Relevant irrelevant regions

**Figure 17:**
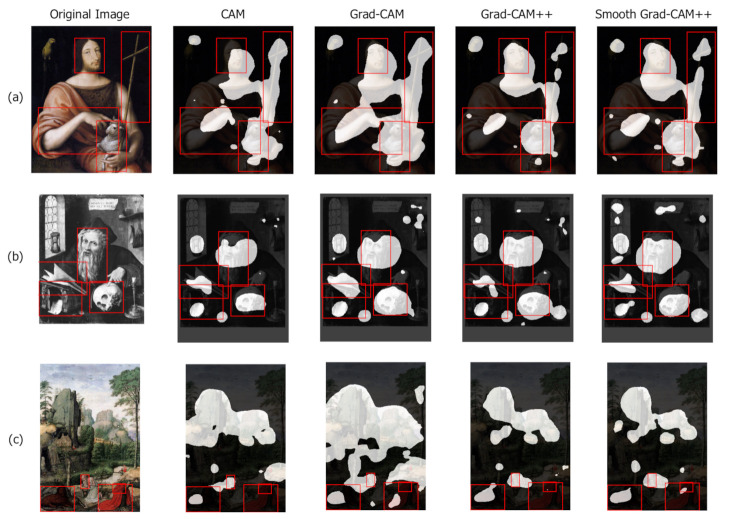
Class activation maps highlighting regions containing relevant iconographic attributes not present in the ground truth: a bird associated with Saint John the Baptist (**a**) an hourglass associated with Saint Jerome (**b**) and the wilderness where Saint Jerome retired (**c**).

An interesting case occurs when the class activation map algorithms focus on an apparently irrelevant area, which instead contains a relevant iconographic attribute not present in the ground truth. Figure 17 illustrates three examples. The painting of Saint John the Baptist (a) (portrait of François I as St John the Baptist, Jean Clouet, 1518) contains an apparently irrelevant area in the top left, which focuses on a bird. This is a less frequent attribute of the Saint that is not listed in the iconographic symbols used to annotate the images but appears in some of the paintings. The same happens with Saint Jerome (b) (Saint Jerome, Albrecht Durer, 1521), where the class activation map algorithms highlight an hourglass, an infrequent symbol present only in a subset of the ArtDL images and not used in the annotation. Finally, another case occurs with the iconography of Saint Jerome (c) (Landscape with St. Jerome, Simon Bening, 1515–1520), where the class activation map algorithms focus on the outdoor environment. This is a well-known symbol associated with the Saint, who retired in the wilderness, but one that is hard to annotate with bounding boxes and thus purposely excluded from the ground truth.

Confusion with unknown co-occurring class

**Figure 18:**
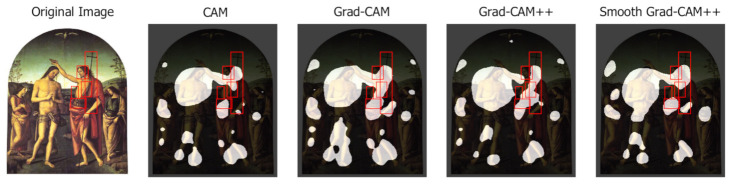
Class activation maps with confusion between Saint John the Baptist and Jesus Christ.

Figure 18 (Baptism of Christ, Pietro Perugino, 1510) presents an example in which all analyzed variants make confusion between Saint John the Baptist and Jesus Christ. The latter is an Iconclass category too, but not one represented in the ArtDL dataset. Given the prevalence of paintings depicting Saint John the Baptist in the act of baptizing Christ over those where the Saint occurs alone, the CAM output highlights both the figures. This ambiguity would reduce if the dataset were annotated with the Iconclass category for Jesus.

#### Bounding Box Generation

The goal of the presented work is to compare the effectiveness of alternative class activation map algorithms in isolating the salient regions of artwork images that have the greatest impact for the attribution of a specific iconography class. The capacity of a class activation map algorithm to identify precisely the areas of an image that correspond to the whole Saint or to one of the iconographic symbols that characterize him/her can help build a training set for the object detection task. The class activation map can be used as a replacement of the manual annotations necessary for creating a detection training set by computing the smallest bounding boxes that comprise the foreground area and using such automatically generated annotations for training an object detector. This approach is known as weakly supervised object detection and is an active research area [48]. To investigate the potential of the class activation maps to support weakly supervised object detection, the region proposals obtained by drawing bounding boxes around the connected components of the class activation maps have been compared visually with the ground-truth bounding boxes of the iconographic symbols. For completeness, we have also computed the bounding boxes surrounding all the foreground pixels and compared them with manually created bounding boxes surrounding the whole Saints. The candidate region proposals to use as automatic bounding boxes have been identified with the following heuristic procedure.

1. Collect the images on which all the four methods satisfy a minimum quality criterion: for symbol bounding boxes component IoU greater than 0.165 at threshold 0.1 (see Figure 6) and for whole Saint bounding boxes global IoU greater than 0.24 at threshold 0.05 (see Figure 8);
2. Compute the Grad-CAM class activation map of the selected images and apply the corresponding threshold: 0.1 for symbol bounding boxes and 0.05 for whole Saint bounding boxes;
3. Only for symbol boxes: split the class activation maps into connected components. Remove the components whose average activation value is less than half of the average activation value of all components. This step filters out all the foreground pixels with low activation that usually correspond to irrelevant areas (Figure 12);
4. For each Iconclass category, draw one bounding box surrounding each component (symbol bounding boxes) and one bounding box surrounding the entire class activation map (whole Saint bounding boxes).

In the procedure above, Grad-CAM is chosen to compute the candidate symbol and whole Saint bounding boxes, because it has the highest value of the bounding box coverage metrics (together with Smooth Grad-CAM++ ) and covers wider areas, at the same time, focusing on the correct details.

Symbol bounding boxes 

**Figure 19:**
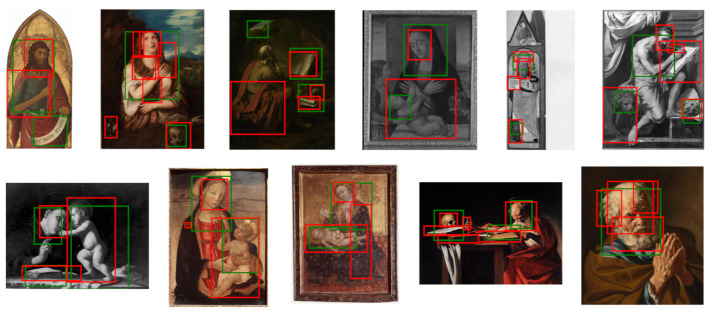
Examples of symbols bounding boxes generated from Grad-CAM (green) and manually annotated (red).

Figure 19 presents some examples of the computed symbol bounding boxes (green) compared with the ground-truth bounding boxes (red). The proposed procedure is able to generate boxes that in many cases correctly highlight and distinguish the most important iconographic symbols present in the images. When the symbols are grouped in a small area (e.g., the bushy hair and beard of Saint Peter), the procedure tends to generate one component that covers all of them, thus creating only one bounding box. Sometimes, elements in the image that have not been manually annotated in the ground truth are correctly detected (e.g., the scroll in the hand of Saint John the Baptist in the first painting of Figure 19).

Whole Saint bounding boxes 

**Figure 20:**
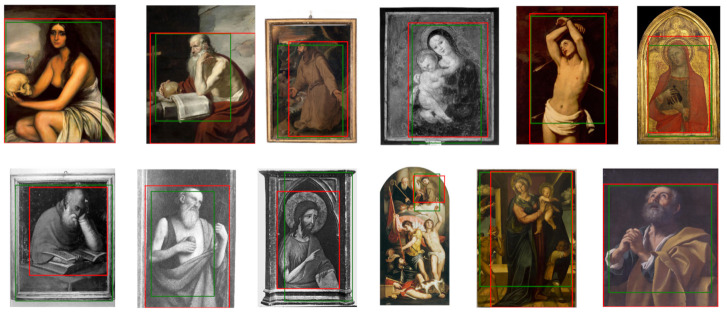
Examples of Saints bounding boxes generated from Grad-CAM (green) and manually annotated (red).

Figure 20 illustrates some examples of computed whole Saint bounding boxes (green) compared with the ground-truth boxes (red). The automatically generated bounding boxes localize almost entirely the Saint’s figure and include only very small irrelevant areas.

Figure 19 and Figure 20 show that the simple procedure for processing class activation map outputs is sufficient to generate good quality bounding boxes that can act as a proxy to the ground truth for training a fully supervised object detector.

Quantitative evaluation of whole Saint bounding boxes

**Figure 21:**
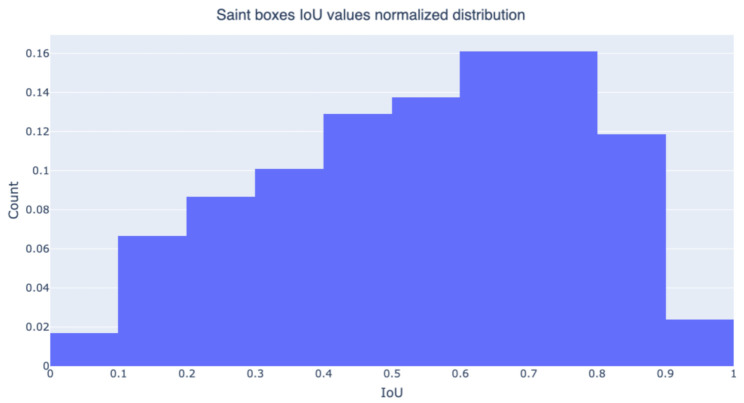
Normalized distribution of IoU values between whole-Saint Grad-CAM estimated bounding boxes and ground-truth bounding boxes.

For the whole Saint case, each estimated bounding box can be labeled with the iconography class of the corresponding Saint portrayed in the image. In this way, it is possible to quantify the coincidence between the bounding box of the ground truth and the bounding box computed from the class activation map. For this purpose, three object detection metrics have been computed: the average IoU value between the GT and the estimated bounding boxes, mean Average Precision and GT-known Loc. The latter is used in several works ([49,50,51]) to evaluate the localization accuracy of object detectors and is defined as the percentage of correct bounding boxes. A bounding box is considered correct only when the IoU between the GT box (for a specific class) and the estimated box (for the same class) is greater than 0.5. Results are reported in Table 3: Grad-CAM confirms as the method with the best performances, Smooth-Grad-CAM++ yields similar results, and CAM is the worst performing method in all the computed metrics. Grad-CAM produces bounding boxes that on average have 0.55 IoU with the GT boxes and the GT-known Loc metric shows that ∼61% of those boxes have an IoU value greater than 0.5. Figure 21 presents the normalized distribution of IoU values for Grad-CAM. We can observe that ∼83% of the generated boxes have an IoU value greater than 0.3 and that most values are in the range between 0.4 and 0.9, with ∼12% having an IoU greater than 0.9. Table 4 shows the mAP values obtained with GradCAM on the ten ArtDL classes.

The whole Saint estimated bounding boxes appear to be suitable for creating the pseudo ground truth for training an object detector with the weakly supervised approach. Two observations motivate the viability of Grad-CAM for this purpose. As in the GT-known Loc metrics, the goodness of an object detection is usually evaluated with a minimal IoU threshold of 0.5 and the boxes generated automatically with Grad-CAM obtain 0.55 IoU on average, which suggests that the automatically estimated bounding boxes have a quality similar to the bounding boxes produced by a fully supervised object detector, albeit inferior to the quality of the bounding boxes created by humans. Grad-CAM, which is designed to be an interpretability technique, can be used also to estimate bounding boxes that reach 31.6% mAP on cultural heritage data without any optimization. This finding compares well with the fact that methods designed and optimized specifically for weakly supervised object detection reach values around 14% on artworks datasets similar to ArtDL [10,52]. For this reason, simple and generic techniques such as Grad-CAM, which can localize multiple Saint instances and even multiple characteristic features, are a promising starting point for advancing weakly supervised object detection studies in the cultural heritage domain.

## 5. Conclusions and Future Work

This work has presented a comparative study about the effectiveness of class activation maps as a tool for explaining of how a CNN-based classifier recognizes the Iconclass categories present in images portraying Christian Saints. The symbols relevant to the identification of the Saints were annotated with bounding boxes and the output of the class activation maps algorithms were compared to the ground truth using four metrics. The analysis shows that Grad-CAM achieves better results in terms of global IoU and covered bounding boxes and Smooth Grad- CAM++ scores best in the component IoU thanks to its precision in delineating individual small size symbols. The irrelevant attention metric promotes the original CAM algorithm as the best approach, but the low component IoU and box coverage complement such an evaluation showing that CAM covers too small areas. While for natural images Smooth Grad-CAM++ outperforms the other three algorithms [18], in our use case Grad-CAM is the method of choice for deriving the bounding boxes from class activation maps necessary to train a weakly supervised detector.

Future work will concentrate on the comparison of other activation mapping techniques [50,51,53,54]. In particular, [50,51] are based on the re-training of the network, an approach quite different from the currently analyzed alternatives. The results of the CAMs algorithms selection will be used to pursue the ultimate goal of our research, which is to use the output of class activation maps to create training datasets for weakly supervised iconographic symbol detection and segmentation. The implementation of an automated system for iconographic analysis of artworks could promote the development of educational applications for art history experts and students. Finally, another future research path consists in addressing more complex Iconclass categories involving complex scenes (e.g., the crucifixion, the nativity, the visitation of the magi, etc.) and in exploring the iconography of other cultures.

## Figures and Tables

**Table 1:** Iconclass categories and symbols associated with them.

Iconclass Category	Symbols
**Anthony of Padua**	Baby Jesus, bread, book, lily, face, cloth
**Dominic**	Rosary, star, dog with a torch, face, cloth
**Francis of Assisi**	Franciscan cloth, wolf, birds, fish, skull, stigmata, face, cloth
**Jerome**	Hermitage, lion, cardinal’s galero, cardinal vest, cross, skull, book, writing material, stone in hand, face, cloth
**John the Baptist**	Lamb, head on platter, animal skin, pointing at Christ, pointing at lamb, cross, face, cloth
**Mary Magdalene**	Ointment jar, long hair, washing Christ’s feet, skull, crucifix, red egg, face, cloth
**Paul**	Sword, book, scroll, horse, beard, balding head, face, cloth
**Peter**	Keys, boat, fish, rooster, pallium, papal vest, inverted cross, book, scroll, bushy beard, bushy hair, face, cloth
**Sebastian**	Arrows, crown, face, cloth
**Virgin Mary**	Baby Jesus, rose, lily, heart, seven swords, crown of stars, serpent, rosary, blue robe, sun and moon, face, cloth, crown

**Table 2:** Symbol and bounding box distribution.

Iconclass Category	Symbol Classes	Symbol Bounding Boxes
**Anthony of Padua**	6	83
**Dominic**	4	59
**Francis of Assisi**	5	295
**Jerome**	11	434
**John the Baptist**	5	231
**Mary Magdalene**	5	283
**Paul**	6	132
**Peter**	9	408
**Sebastian**	3	267
**Virgin Mary**	7	695

**Table 3:** Average IoU, GT-Known accuracy and mAP values for the whole Saint bounding boxes estimated with the four analyzed class activation map techniques. The values are calculated with an activation threshold equal to 0.05.

Method	Average IoU	GT-Known Loc (%)	mAP (at IoU ≥0.5)
CAM	0.489	49.70	0.206
GradCAM	0.551	61.20	0.316
GradCAM++	0.529	59.88	0.292
Smooth-GradCAM++	0.544	61.18	0.307

**Table 4:** Mean Average Precision (mAP) values for each class of the ArtDL dataset. Bounding boxes are estimated with GradCAM.

Anthony	John	Paul	Francis	Magdalene	Jerome	Dominic	Virgin	Peter	Sebastian
0.076	0.289	0.173	0.33	0.616	0.228	0.142	0.442	0.399	0.468

## Data Availability

Publicly available datasets were analyzed in this study. This data can be found here: http://www.artdl.org (accessed on 29 June 2021).
